# Hippocampal SIRT1 signaling mediates the ameliorative effect of treadmill exercise on anxiety- and depression-like behavior in APP/PS1 mice

**DOI:** 10.3389/fnagi.2024.1489214

**Published:** 2024-12-18

**Authors:** Yarong Wang, Rongxing Zhang, Yumin Jiang, Jingwen Liao, Lianwei Mu, Min Hu

**Affiliations:** ^1^Scientific Research Center, Guangzhou Sport University, Guangzhou, China; ^2^Guangdong Provincial Key Laboratory of Physical Activity and Health Promotion, Guangzhou Sport University, Guangzhou, China

**Keywords:** SIRT1, treadmill exercise, anxiety-and depression-like behaviors, mitochondrial, APP/PS1 mice

## Abstract

**Objective:**

Anxiety and depression-like symptoms occur in the early stages of Alzheimer’s disease. Hippocampal Sirtuin 1 (SIRT1) signaling mediates anxiety- and depression-like behavior. Exercise training improves anxiety and depression-like behavior in various disease models, such as the rat chronic restraint stress model, rat model of posttraumatic stress disorder, and rat model of fetal alcohol spectrum disorders. Here, we aimed to investigate whether exercise ameliorates anxiety- and depression like behaviors in APP/PS1 mice and explore the potential mechanisms.

**Methods:**

After eight weeks of exercise intervention, we assessed anxiety- and depression-like behaviors in Alzheimer’s disease (AD) model mice. We then measured the levels of SIRT1, peroxisome proliferator-activated receptor gamma coactivator-1 alpha (PGC1α), nuclear respiratory factor 1 (NRF1), mitochondrial transcription factor A (TFAM), and mitochondrial biogenesis (CO2, ATP6, and mitochondrial content) using immunofluorescence, reverse transcription-quantitative real-time PCR, and transmission electron microscopy. Finally, we investigated the effects of pharmacological activation of SIRT1 on anxiety- and depression-like behaviors, the SIRT1/PGC-1α/NRF1/TFAM signaling axis, and mitochondrial biogenesis.

**Results:**

We first observed that treadmill exercise improved anxiety- and depression-like behaviors in six-month-old APP/PS1 mice and increased SIRT1 levels in the hippocampus. Pharmacological activation of hippocampal SIRT1 function also reduced anxiety and depression-like behaviors in APP/PS1 mice. Meanwhile, both treadmill exercise and pharmacological activation of hippocampal SIRT1 increased the levels of PGC1α, NRF1, TFAM, and enhanced mitochondrial biogenesis (CO2, ATP6, or mitochondrial content) in the hippocampus of APP/PS1 mice.

**Conclusion:**

These findings reveal that treadmill exercise reduces anxiety- and depression-like behaviors in six-month-old APP/PS1 mice by enhancing the SIRT1-dependent PGC-1α/NRF1/TFAM axis, promoting mitochondrial biogenesis in the hippocampus.

## 1 Introduction

Alzheimer’s disease (AD) is the most common chronic neurodegenerative disease, pathologically characterized by the extracellular deposition of *β*-amyloid (Aβ) and intracellular neurofibrillary tangles in the brain. Pathological Aβ and tau proteins cause the loss of synaptic function, activation of microglia and astrocytes, mitochondrial damage, and neuronal death. This ultimately leads to clinical symptoms involving progressive cognitive dysfunction and memory impairment of AD. The core symptoms of AD are cognitive dysfunction and memory impairment. However, neuropsychiatric symptoms such as anxiety and depression are also commonly observed during the early and middle stages of AD (Gao et al., 2018).

In AD, the prevalence of anxiety ranges from 9.4% in the preclinical phase to 39% in cases of mild to severe AD (Botto et al., 2022; Zhao et al., 2016). Additionally, the prevalence of depression in individuals with mild-to-moderate AD ranges from 14.8 to 40% (Asmer et al., 2018; Botto et al., 2022). Indeed, research has demonstrated that behaviors resembling anxiety and depression can serve as predictive indicators and causal elements that expedite the progression of AD (Peters and Lyketsos, 2015). Timely intervention for symptoms resembling depression may mitigate subsequent memory impairment, consequently lowering the likelihood of developing dementia (Almeida et al., 2017; Peters and Lyketsos, 2015). Although there are currently some drugs (galantamine, rivastigmine, and donepezil) that can be used to treat Alzheimer’s disease, they have relatively small average overall effects and do not alter the course of the underlying neurodegenerative process. Previous studies by us and others have demonstrated that treadmill exercise can effectively ameliorate spatial learning and memory in an AD mouse model by reducing pathological Aβ production and abnormal tau phosphorylation (Mu et al., 2022a,b; Xu et al., 2022). Meanwhile, both human and animal studies have found that exercise effectively alleviates and prevents anxiety- and depression-like behavior (Gumus et al., 2022; Ji et al., 2022). However, it remains unclear whether exercise improves anxiety- and depression-like behaviors in the early stages of AD. The present study will examine the effects of 8 weeks of treadmill exercise on anxiety- and depression-like behaviors in six-month-old APP/PS1 mice. Subsequently, the potential mechanisms involved are examined.

Mammalian sirtuins (SIRTs) are histone and protein deacetylases that depend on NAD^+^ and are crucial in various cellular processes such as the cell-division cycle, transcriptional repression, recombination, DNA repair, energy homeostasis, and responses to cellular stress (Alves-Fernandes and Jasiulionis, 2019; Carafa et al., 2016; Li, 2013). Dysfunction in SIRT is intricately linked to the pathophysiology of neurodegenerative and psychiatric disorders, including Alzheimer’s disease, anxiety, and depression (Cao et al., 2018; Lu et al., 2018). It has been consistently demonstrated that the levels and activity of Sirtuin 1 (SIRT1) in the cerebral cortex and hippocampus of patients with Alzheimer’s disease (AD) are diminished (Cao et al., 2018; Julien et al., 2009). This decrease correlates with the accumulation of AD pathology and the progression of the disease. Meanwhile, animal experiments have revealed that chronic stress leads to a reduction in SIRT1 expression in the dentate gyrus (DG) of the hippocampus and the bed nucleus of the stria terminalis (BNST) (Abe-Higuchi et al., 2016; Hu et al., 2023; Liu et al., 2019). However, pharmacological activation or local overexpression of SIRT1 in the hippocampus or BNST can reverse chronic stress-induced anxiety- and depression-like behaviors (Abe-Higuchi et al., 2016; Hu et al., 2023; Liu et al., 2019). Therefore, SIRT1 signaling may mediate anxiety- and depression-like behaviors in AD model mice. Studies have shown that regulating mitochondrial structure and function can significantly improve pathology and cognitive function in APP/PS1 mice (Stojakovic et al., 2021; Trushina et al., 2023). SIRT1 has been demonstrated to promote mitochondrial biogenesis through the activation of peroxisome proliferator-activated receptor gamma coactivator-1 alpha (PGC-1a) (Amat et al., 2009). PGC-1α is a transcriptional coactivator that activates the nuclear respiratory factors 1 (NRF1) and mitochondrial transcription factor A (TFAM) to regulate mitochondrial DNA replication and gene transcription (Cardanho-Ramos and Morais, 2021; Zhao et al., 2020). Therefore, the SIRT1-dependent PGC-1α/NRF1/TFAM signaling pathway, which mediates mitochondrial biogenesis, plays a significant role in the intervention of anxiety and depression-like behaviors.

In the present study, we further examine whether 8 weeks of treadmill exercise affects SIRT1 expression, the PGC-1α/NRF1/TFAM axis, and mitochondrial biogenesis (specifically, the level of mtDNA-encoded complex IV subunit CO2 and Complex V subunit ATP6, as well as the number of mitochondria) in the hippocampus of APP/PS1 mice. We found that APP/PS1 mice exhibited anxiety- and depression-like behaviors, and exercise alleviated these behaviors in APP/PS1 mice. Pharmacological activation of SIRT1 also improves anxiety- and depression-like behaviors in APP/PS1 mice. In addition, treadmill exercise increased the levels of the SIRT1/PGC-1α/NRF1/TFAM axis and improved mitochondrial biogenesis in the hippocampus of APP/PS1 mice. Overall, our findings suggest that hippocampal SIRT1 signaling mediates the ameliorative effect of exercise on anxiety- and depression-like behaviors in APP/PS1 mice. These findings offer a new perspective on understanding the mechanisms by which exercise helps alleviate anxiety- and depression-like behaviors in APP/PS1 mice.

## 2 Materials and methods

### 2.1 Animals

Male APP/PS1 transgenic mice and wild-type mice (WT) were purchased from the Guangdong Medical Laboratory Animal Center. The mice were housed in a room maintained at a controlled temperature of 23 ± 1°C and humidity ranging from 40 to 60%, with a 12-h light–dark cycle (lights on at 7:00 a.m.). Meanwhile, mice in all groups had free access to pelleted food and water bottles. All animal maintenance and use were in accordance with protocols approved by the Ethical Committee of Guangzhou Sport University (2023DWL-31). All reasonable efforts have been made to minimize animal suffering.

### 2.2 Treadmill exercise protocol

Mice were randomly separated into four groups: wild-type sedentary group (WT-Sed; *n* = 6), wild-type exercise group (WT-Ex; *n* = 6), APP/PS1 sedentary group (AP-Sed; *n* = 6), and APP/PS1 exercise group (AP-Ex; *n* = 6). Mice in the exercise groups were trained on the treadmill for 8 weeks, starting at 4 months of age and ending at 6 months of age. Mice in the sedentary groups were placed on the treadmill without running for the same duration as the exercise groups. First, the exercise groups were allowed to adapt to treadmill running for 30 min on three consecutive days (first day at 5 m/min; second and third days at 10 m/min). Then, the mice were subjected to a treadmill exercise protocol at a speed of 12 m/min on a 0° slope for 10 min, followed by 15 m/min for 50 min. In total, mice were trained for 1 h per day, 5 days per week, for a total of 8 weeks. We first detected the anxiety- and depression-like behaviors in mice. The hippocampal tissue from each mouse was subsequently collected. One hemisphere from each mouse was fixed for immunofluorescence and transmission electron microscopy (TEM) analysis. The hippocampus from the remaining hemisphere was analyzed for mRNA expression using reverse transcription-quantitative real-time PCR (RT-qPCR).

### 2.3 Drug administration

Resveratrol and SRT2104 are small-molecule activators of SIRT1 that have demonstrated good tolerability and safety in both animal and human studies (Chang et al., 2024; Hoffmann et al., 2013; Hubbard and Sinclair, 2014). The dosage of Resveratrol and SRT2104 used for intraperitoneal (i.p.) injection was determined based on previous reports (Abe-Higuchi et al., 2016; Miyaji et al., 2021; Park and Pezzuto, 2015). Resveratrol (HY-16561) and SRT2104 (HY-15262) were purchased from MedChemExpress (State of New Jersey, United States). Resveratrol or SRT2104 was first dissolved in DMSO (final concentration 5%), then further mixed with 40% PEG300, 5% Tween-80, and 55% sterile saline sequentially with gentle heating. In this study, we investigate the ameliorative effect of resveratrol and SRT2104 on anxiety- and depression-like behaviors in APP/PS1 mice. In the treatment groups (*n* = 6 in each group), resveratrol (RSV, 200 mg/kg/day, 0.2 mL) or SRT2104 (SRT, 10 mg/kg/day, 0.2 mL) was intraperitoneally injected twice a week from week 16 to week 24 (Abe-Higuchi et al., 2016; Miyaji et al., 2021; Park and Pezzuto, 2015). The APP/PS1 control groups (*n* = 6) were injected intraperitoneally with the same volume of solvent (Vehicle, 0.2 mL), and the injection time and frequency were identical to those of the resveratrol or SRT2104 injections. After 8 weeks of treatment, the anxiety- and depression-like behaviors testing was conducted on mice. Then, we anesthetize the mice to collect hippocampal tissue and detect histological indicators.

### 2.4 Test of anxiety-like behavior

Mice were gently handled for 10 min each day for 3 days before the start of behavioral testing to habituate them to handling. All groups of mice underwent the same behavioral test battery. Each mouse was used only once during each behavioral task. All testing was conducted during the light phase of the light/dark cycle, between 9:00 a.m. and 5:00 p.m. The apparatus was cleaned with 75% ethanol after each test.

#### 2.4.1 Open field test

The open field test is one of the most common and traditional tests used to measure anxiety and locomotor activity in rodents. The open field apparatus is a square arena that consists of white plastic panels (50 cm long; 50 cm wide; 30 cm high). The square arena was divided into a center zone (25 × 25 cm) and a peripheral zone. At the start of the test, mice were placed individually at the corner of the open field and allowed to freely explore the arena during a 5-min test session (Vickstrom et al., 2021). Animal activity and movement trajectory were recorded using an automated video-tracking system. The total distance traveled in the arena was measured as an index of locomotion. Time spent in the center zone, number of center entries, and percentage of distance in the center zone were measured as an index of anxiety.

#### 2.4.2 Marble burying test

The marble burying test was performed in a 30 cm × 18 cm × 12 cm cage filled with corn cobs evenly distributed in a 7 cm deep layer. Twenty-four glass marbles (14 mm in diameter) were placed at equal distances on top of the bedding. Each mouse was placed in a corner of the cage and allowed to freely explore the cage for 20 min (Vickstrom et al., 2021). Afterward, the mice were transferred back to their home cages, and the buried marbles were counted. Buried marbles are defined as marbles that are covered at least two-thirds (2/3) of their size by corn cobs. Studies have found that anxiolytics decrease the number of marbles buried by mice (Li et al., 2006).

#### 2.4.3 Elevated plus maze

The elevated plus maze apparatus consists of two open arms (30 × 5 × 1 cm) positioned across from each other and perpendicular to two closed arms (30 × 5 × 15 cm) that are connected by a central platform (5 × 5 cm). The plus maze is elevated 50 cm above the floor. Mice were placed in the center of the plus maze facing a closed arm and allowed to freely explore the maze for 5 min (Vickstrom et al., 2021). The animal behaviors were recorded by a video camera positioned above the plus maze. Entry or exit to the open arms was defined as having all four paws either inside or outside of the open arms area. The time spent in the open arms and the number of entries into the open arms were quantified to assess the anxiety behavior of mice.

#### 2.4.4 Novelty-suppressed feeding

The novelty-suppressed feeding test was conducted as previously described with minor modifications (Vickstrom et al., 2021). The mice were food-deprived for 24 h but had free access to water before the test. During the test, mice were placed in one corner of the plastic box (50 cm long, 50 cm wide, 30 cm high) where 2–3 food pellets (regular chow) were positioned on a piece of white filter paper (12.5 cm in diameter) in the center of the box. The latency of the mice to consume familiar food was measured within 6 min. Immediately after the mouse began eating the food pellets, it was transferred to the home cage. Feeding was defined as biting the food using the forepaws, not simply sniffing or touching the food.

### 2.5 Tests of depression-like behavior

#### 2.5.1 Splash test

The splash test is based on the active self-grooming behavior of mice and is used to evaluate their depression state (Becker et al., 2021). Mice are placed in a new cage (15 cm × 30 cm × 20 cm) with a 1 cm layer of corn cobs. Additionally, a 10% sucrose solution (750 μL) is sprayed on their dorsal coat. The animal behaviors were recorded for 5 min by a video camera positioned above the cage and later analyzed for the latency to the first grooming, the time spent engaged in this behavior, and its frequency (Calpe-López et al., 2019). Mice developing depression-like symptoms spent less time grooming and had increased latency.

#### 2.5.2 Sucrose preference test

The sucrose preference test was performed to investigate depression-like behavior based on the animal’s natural preference for sweets (Wu et al., 2020). Prior to testing, mice were individually housed for 24 h with two drinking bottles of tap water. Following this acclimation, mice were deprived of food and water for 8 h, followed by free choice of either a 1% sucrose solution or tap water for 16 h (Vickstrom et al., 2021). The intake of sucrose solution and tap water was measured by weighing the bottle. The sucrose preference index (%) was calculated as the amount of sucrose solution consumed divided by the total amount of solution consumed.

#### 2.5.3 Tail suspension test

The tail suspension test was conducted based on a previous study (Eltokhi et al., 2021). Mice were suspended 50 cm above the floor by adhesive tape placed approximately 1 cm from the tip of their tails. The mice initially tried to escape from tail suspension by engaging in vigorous movements and then became immobile after a few minutes. The animal behaviors were recorded by a video camera positioned beside the mice. During this test, immobility time and the latency to first immobility were recorded during the 6-min period. An increased duration of immobility or a reduced latency to first immobility is indicative of a depression-like phenotype.

#### 2.5.4 Forced swim test

The forced swim test is the most widely used method for assessing depression-like behavior in mice. Mice were gently lowered into a clear plastic cylinder (height 30 cm, diameter 10 cm) filled with water (30 ± 1°C) to a depth of 18 cm for 6 min (Vickstrom et al., 2021). The animal behaviors were recorded by a video camera positioned on the side of the plastic cylinder. Immobility time between 2 and 6 min was recorded. Immobility is defined as the cessation of all movements (e.g., climbing, swimming) except those necessary for the mouse to keep its head above water (i.e., floating). The time of physical immobility can be considered a measure of behavioral despair in mice.

### 2.6 Reverse transcription-quantitative real-time PCR

Mice were deeply anesthetized with isoflurane inhalation and then decapitated. The whole hippocampal samples were rapidly removed and stored at −80°C until used. Total RNA was isolated from the snap-frozen whole hippocampi using TRIzol reagent and then converted into cDNA with a RevertAid First Strand cDNA Synthesis Kit. The cDNA obtained was mixed with gene-specific primers (Table 1) for real-time PCR in a StepOnePlus instrument. The primers originated from Servicebio (China). Detailed reaction conditions were as follows: 95°C for 10 min, 40 cycles of 95°C for 15 s, and 60°C for 1 min. The relative mRNA amount was determined by the 2^−ΔΔCt^ method and normalized with GAPDH expression. Primer sequences used for quantitative RT-qPCR are shown in Table 1.

**Table 1:** Primer sequences used for quantitative RT-qPCR.

Primer	Forward sequence	Reverse sequence
SIRT1	CGAGGTCCATATACTTTTGTTCAG	GCGTCATATCATCCAGCTCAG
PGC1α	CTGGGTGGATTGAAGTGGTGTA	AGTGGTCACGGCTCCATCTGT
NRF1	CGCAGCACCTTTGGAGAATG	CCCCGACCTGTGGAATACTTG
TFAM	CTGTGGAGGGAGCTACCAGAAG	GCTGACTTGGAGTTAGCTGCTCT
GAPDH	CCTCGTCCCGTAGACAAAATG	TGAGGTCAATGAAGGGGTCGT
ATP6	TCACTTGCCCACTTCCTTCCA	GGACTGCTAATGCCATTGGTTG
CO2	ATAGACGAAATCAACAACCCCG	GGATTGGAAGTTCTATTGGCAG

### 2.7 Immunofluorescence staining

Immunofluorescence staining was performed according to published protocols with minor modifications (Mu et al., 2022a,b). Mice were fully anesthetized with isoflurane inhalation and then decapitated. The brain was then removed and immersed in a 4% paraformaldehyde solution for 24 h at 4°C before being dehydrated in increasing concentrations of sucrose (20 and 30%) in 0.1 M PBS. Coronal sections of the hippocampus (20 μm) were cut using a Leica cryostat. Tissue sections are mounted onto glass slides and then subjected to immunofluorescence staining. Hippocampal sections were washed with PBS and then incubated with a blocking buffer (5% normal goat serum +0.3% Triton X-100 in PBS) at room temperature for 2 h. The hippocampal sections were then incubated with primary antibodies (SIRT1, 1:2000, GB11512, Servicebio, China) for 24 h at 4°C. Hippocampus sections were then incubated with secondary antibodies (Alexa Fluor 594, 1:5000, GB28301, Servicebio, China) for 4 h at room temperature in the dark. Finally, the sections were cover-slipped with the glycerol mounting medium containing DAPI and imaged using a Nikon Eclipse TE-2000 U confocal microscope. SIRT1 fluorescence intensity was calculated using Image J and normalized to the control group.

### 2.8 Transmission electron microscopy examination

Transmission electron microscopy examinations are based on our previous study (Mu et al., 2022a,b). Briefly, mice were fully anesthetized with isoflurane inhalation and then decapitated. The hippocampal tissue was cut into 1 mm^2^ pieces and immersed in a solution containing 4% paraformaldehyde and 2.5% glutaraldehyde in 0.1 M phosphate buffer for 24 h. The hippocampus was stained with 1% osmium tetroxide and then dehydrated in a graded series of acetone. Tissues were cut into ultrathin sections, stained with uranyl acetate and lead citrate, and examined using a Hitachi H-7100 electron microscope. We collected 10–15 sections from the hippocampus of 6 mice in each experimental group. Photographs of random positions from these specimens were taken at ×10,000 magnification. The number of mitochondria per 10 μm^2^ was counted.

### 2.9 Statistics

Animals were excluded from the analysis if they failed to reach the criterion at test stages or after they developed age-related reduced visual ability. The data involved in the study are presented as the mean ± SEM. Data analysis and visualization were performed using SigmaPlot 14.0. Data analysis was blind to the genotypes and treatment history of the mice. Statistical analysis of the results was performed using two-way ANOVA or one-way ANOVA, followed by Tukey’s *post hoc* analysis. *Post-hoc* analyses were performed only when ANOVA yielded a significant main effect or a significant interaction between the two factors. A value of *p* < 0.05 was considered to indicate a statistically significant difference.

## 3 Results

### 3.1 Treadmill exercise ameliorates anxiety-like behavior of APP/PS1 mice

**Figure 1:**
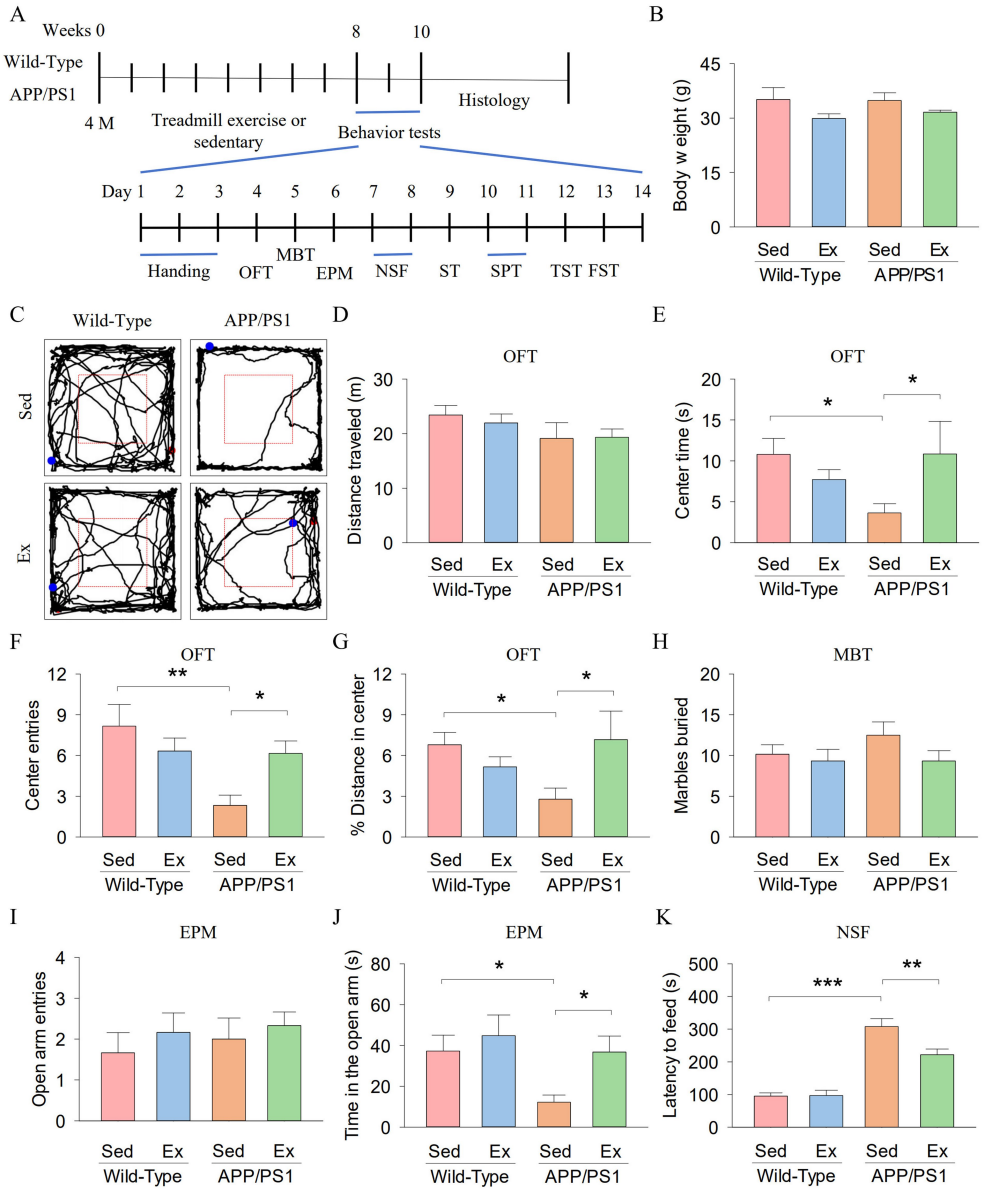
Treadmill exercise ameliorates anxiety-like behavior of APP/PS1 mice. **(A)** The experimental design of the animal study. **(B)** Body weight of mice. **(C)** Representative trajectories of mice in the OFT. **(D–G)** The distance traveled **(D)**, the center time **(E)**, center entries **(F)**, and % distance in center **(G)** in the OFT for each group of mice. **(H)** Number of marbles buried by mice in the MBT. **(I,J)** The open arm entries **(I)** and time in the open arm **(J)** by mice in each experimental group during the EPM. **(K)** The latency to feed of mice in the NSF. n = six per group. Data are presented as mean ± SEM, two way ANOVA; *p < 0.05, **p < 0.01, ***p < 0.001.

We first investigated whether six-month-old APP/PS1 mice exhibited anxiety-like behavior and whether treadmill exercise could ameliorate this behavior in the mice. Wild-type (WT) and APP/PS1 (AP) mice underwent 8 weeks of treadmill exercise (Ex) or non-exercise control (Sed) treatment starting at 4 months of age (2 × 2 factorial design: genotype vs. exercise). After the 8-week training, the open field test (OFT), marble burying test (MBT), elevated plus maze (EPM), novelty-suppressed feeding (NSF) were used to investigate anxiety-like behavior (Figure 1A). The mouse weight was not different among the four groups (Figure 1B). Figure 1C shows representative trajectories of mice in the OFT. In the OFT, total distance traveled did not show significant differences among the four groups (Figure 1D). Two-way ANOVA showed that genotype and exercise had no significant main effect on the center time (genotype: *F*_1,23_ = 0.7, *p* = 0.404; exercise: *F*_1,23_ = 0.8, *p* = 0.391; Figure 1E), center entries (exercise: *F*_1,23_ = 0.8, *p* = 0.376; Figure 1F), and % distance in center (genotype: *F*_1,23_ = 0.6, *p* = 0.446; exercise: *F*_1,23_ = 1.2, *p* = 0.288; Figure 1G), but there was a significant interaction between genotype and exercise on the center time (*F*_1,23_ = 4.8, *p* = 0.041), center entries (genotype: *F*_1,23_ = 7.4, *p* = 0.013; genotype × exercise interaction: *F*_1,23_ = 6.6, *p* = 0.018), and % distance in center (*F*_1,23_ = 5.6, *p* = 0.028). Tukey’s *post-hoc* tests indicated that the center time (*p* = 0.045; Figure 1E), center entries (*p* = 0.001; Figure 1F), and % distance in center (*p* = 0.038; Figure 1G) were significantly decreased in the AP-Sed group compared to the WT-Sed group. Treadmill exercise increased the center time (*p* = 0.043), center entries (*p* = 0.024), and % distance in center (*p* = 0.024) of APP/PS1 transgenic mice.

In the MBT, there were no significant differences in the number of marbles buried among the four groups (Figure 1H). In the EPM, open arm entries did not exhibit significant differences among the four groups (Figure 1I). Two-way ANOVA revealed that genotype and exercise had significant main effects on time in the open arm (genotype: *F*_1,23_ = 4.7, *p* = 0.043; exercise: *F*_1,23_ = 4.4, *p* = 0.049; Figure 1J). However, there was no significant interaction between genotype and exercise on time in the open arm (*F*_1,23_ = 1.2, *p* = 0.278). Tukey’s *post-hoc* tests indicated that time in the open arm (*p* = 0.032; Figure 1J) was significantly decreased in the AP-Sed group compared to the WT-Sed group. Treadmill exercise increased time in the open arm (*p* = 0.035) of APP/PS1 transgenic mice.

In the NSF, two-way ANOVA revealed significant effects of genotype and exercise on the latency to feed (genotype: *F*_1,23_ = 93.5, *p* < 0.001; exercise: *F*_1,23_ = 58, *p* = 0.026; genotype *×* exercise interaction: *F*_1,23_ = 6.3, *p* = 0.021; Figure 1K). Tukey’s *post-hoc* tests indicated that latency to feed (*p* < 0.001; Figure 1J) was significantly decreased in the AP-Sed group compared to the WT-Sed group. Treadmill exercise increased time in the open arm (*p* = 0.003) of APP/PS1 transgenic mice. Together, these data suggest that six-month-old APP/PS1 mice exhibited anxiety-like behavior, and treadmill exercise alleviated this behavior in the APP/PS1 mice.

### 3.2 Treadmill exercise ameliorates depression-like behavior of APP/PS1 mice

**Figure 2:**
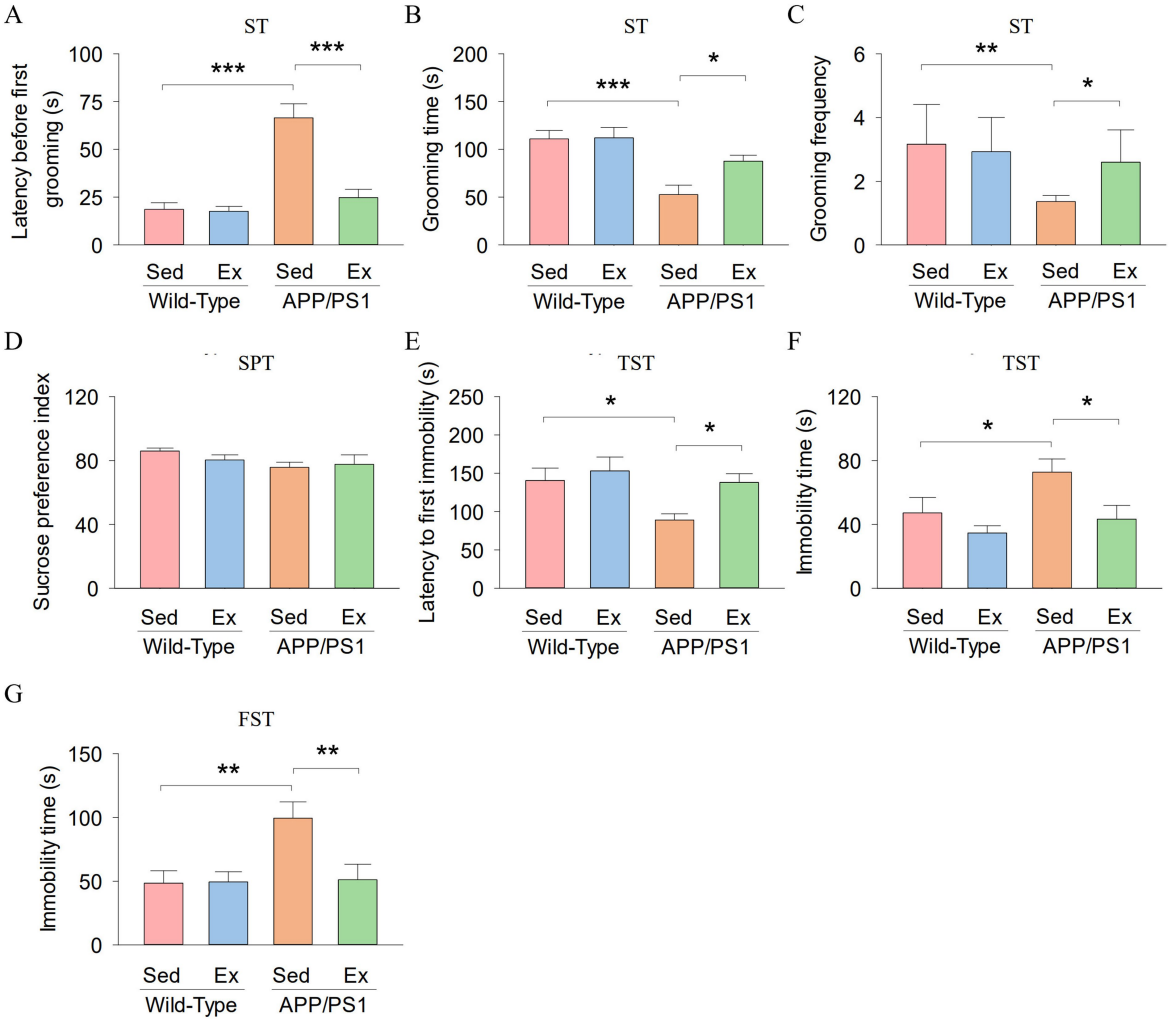
Treadmill exercise ameliorates depression-like behavior of APP/PS1 mice. **(A-C)** The latency before first grooming **(A)**, grooming time **(B)**, and grooming frequency **(C)** in the ST for each group of mice. **(D)** The sucrose preference index of mice in the SPT. **(E,F)** The latency to first immobility **(E)** and immobility time **(F)** by mice in each experimental group during the TST. **(G)** The immobility time by mice in each experimental group during the FST. n = six per group. Data are presented as mean ± SEM, two way ANOVA: *p < 0.05, **p < 0.01, ***p < 0.001.

In addition, we evaluated whether treadmill exercise could ameliorate depression-like behavior in APP/PS1 mice. We assessed depression-like behavior using the splash test (ST), sucrose preference test (SPT), tail suspension test (TST), and the forced swim test (FST). In the ST, two-way ANOVA showed that genotype and exercise had significant effects on the latency before first grooming (genotype: *F*_1,23_ = 32.8, *p* < 0.001; exercise: *F*_1,23_ = 19.6, *p* < 0.001; genotype *×* exercise interaction: *F*_1,23_ = 17.9, *p* < 0.001; Figure 2A). Meanwhile, genotype and exercise had no significant main effect on the grooming time (genotype: *F*_1,23_ = 19.5, *p* < 0.001; exercise: *F*_1,23_ = 3.1, *p* = 0.096; Figure 2B), and grooming frequency (exercise: *F*_1,23_ = 2.1, *p* = 0.167; Figure 2C). However, there was a significant interaction between genotype and exercise on the grooming time (*F*_1,23_ = 5.1, *p* = 0.036), and grooming frequency (genotype: *F*_1,23_ = 9.4, *p* = 0.006; genotype × exercise interaction: *F*_1,23_ = 4.4, *p* = 0.048). Tukey’s *post-hoc* tests indicated that the latency before first grooming (*p* < 0.001; Figure 2A) was significantly decreased in the AP-Sed group compared to the WT-Sed group, but the grooming time (*p* < 0.001; Figure 2B) and grooming frequency (*p* = 0.002; Figure 2C) were significantly increased in the AP-Sed group compared to the WT-Sed group. Treadmill exercise decreased the latency before first grooming (*p* < 0.001) and increased the grooming time (*p* = 0.011) and grooming frequency (*p* = 0.021) of APP/PS1 transgenic mice.

In the SPT, sucrose preference index did not show significant differences among the four groups (Figure 2D). In the TST, two-way ANOVA revealed that genotype and exercise had significant main effects on the latency to first immobility (genotype: *F*_1,23_ = 5.6, *p* = 0.028; exercise: *F*_1,23_ = 4.8, *p* = 0.041; Figure 2E) and immobility time (genotype: *F*_1,23_ = 4.6, *p* = 0.045; exercise: *F*_1,23_ = 6.8, *p* = 0.017; Figure 2F). However, there was no significant interaction between genotype and exercise on the latency to first immobility (*F*_1,23_ = 1.7, *p* = 0.213) and immobility time (*F*_1,23_ = 1.1, *p* = 0.303). Tukey’s *post-hoc* tests indicated that the latency to first immobility (*p* = 0.018; Figure 2E) was significantly decreased in the AP-Sed group compared to the WT-Sed group, but the immobility time (*p* = 0.035; Figure 2F) was significantly increased in the AP-Sed group compared to the WT-Sed group. Treadmill exercise increased the latency to first immobility (*p* = 0.024) and decreased the immobility time (*p* = 0.017) of APP/PS1 transgenic mice.

In the FST, two-way ANOVA revealed that genotype and exercise had significant effects on the immobility time (genotype: *F*_1,23_ = 5.9, *p* = 0.025; exercise: *F*_1,23_ = 4.7, *p* = 0.042; genotype × interaction: *F*_1,23_ = 5.1, *p* = 0.035; Figure 2G). Tukey’s *post-hoc* tests indicated that the immobility time (*p* = 0.004; Figure 2G) was significantly increased in the AP-Sed group compared to the WT-Sed group. Treadmill exercise decreased the immobility time (*p* = 0.005) of APP/PS1 transgenic mice. Together, these data collectively indicate that six-month-old APP/PS1 mice exhibited depression-like behavior, and treadmill exercise alleviated this behavior in the APP/PS1 mice.

### 3.3 Treadmill exercise enhanced the hippocampal SIRT1 expression in APP/PS1 mice

**Figure 3:**
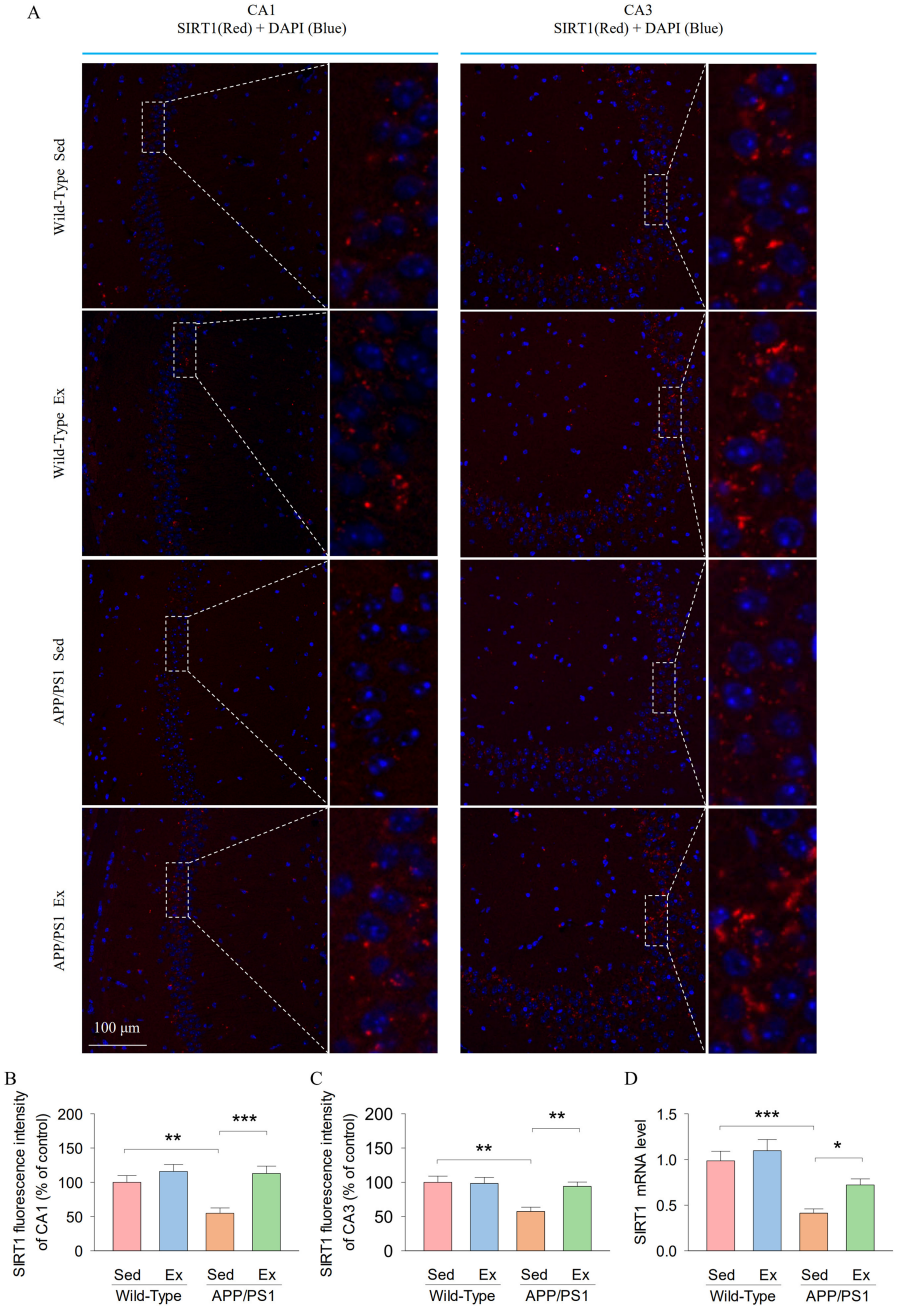
Treadmill exercise enhanced the hippocampal SIRTI expression in APP/PS1 mice. **(A)** Representative microscopy images of SIRT1 in the hippocampal CAI and CA3 regions. Scale bar 100 μm. **(B,C)** Quantitative analysis of SIRT1 fluorescence intensity in the hippocampal CA1 **(B)** and CA3 **(C)** regions. **(D)** The RT-qPCR analysis of SIRT1 in hippocampal tissues from mice. n = sixper group. Data are presented as mean ± SEM, two way ANOVA; *p < 0.05, **p < 0.01, ***p < 0.001.

It has been suggested that hippocampal SIRT1 signaling mediates anxiety- and depression-like behavior (Abe-Higuchi et al., 2016; Kang et al., 2021). We next determined whether treadmill exercise alters the expression of SIRT1 in the hippocampus of APP/PS1 mice using immunofluorescence staining and reverse transcription-quantitative real-time PCR (RT-qPCR). The representative microscopy images of SIRT1 in the hippocampal CA1 and CA3 regions are shown in Figure 3A. Two-way ANOVA revealed that genotype and exercise had significant effects on the SIRT1 fluorescence intensity in both the hippocampal CA1 (genotype: *F*_1,23_ = 6.1, *p* = 0.022; exercise: *F*_1,23_ = 14.7, *p* = 0.001; genotype × exercise interaction: *F*_1,23_ = 4.7, *p* = 0.042; Figure 3B) and CA3 (genotype: *F*_1,23_ = 9.3, *p* = 0.006; exercise: *F*_1,23_ = 5.2, *p* = 0.034; genotype × exercise interaction: *F*_1,23_ = 6.0, *p* = 0.023; Figure 3C). Tukey’s *post-hoc* tests indicated that the SIRT1 fluorescence intensity of the hippocampal CA1 (*p* = 0.004; Figure 3B) and CA3 (*p* = 0.001; Figure 3C) was significantly decreased in the AP-Sed group compared to the WT-Sed group. Treadmill exercise prevented a decrease in the SIRT1 fluorescence intensity in both the hippocampal CA1 (*p* = 0.003) and CA3 (*p* < 0.001) regions in APP/PS1 transgenic mice. Meanwhile, two-way ANOVA revealed that genotype and exercise had significant main effects on the mRNA expression of SIRT1 in the hippocampus (genotype: *F*_1,23_ = 27.8, *p* < 0.001; exercise: *F*_1,23_ = 5.6, *p* = 0.029; Figure 3D). However, there was no significant interaction between genotype and exercise on the mRNA expression of SIRT1 in the hippocampus (*F*_1,23_ = 1.2, *p* = 0.279). Tukey’s *post-hoc* tests indicated that the mRNA expression of SIRT1 (*p* < 0.001; Figure 3D) in the hippocampus was significantly decreased in the AP-Sed group compared to the WT-Sed group. Treadmill exercise prevented a decrease in the mRNA expression of SIRT1 in the hippocampus in APP/PS1 transgenic mice (*p* = 0.024). These data indicate that SIRT1 levels are reduced in the hippocampus of APP/PS1 transgenic mice, but exercise elevated the levels of SIRT1.

### 3.4 Pharmacological activation of SIRT1 ameliorates anxiety- and depression-like behavior of APP/PS1 mice

**Figure 4:**
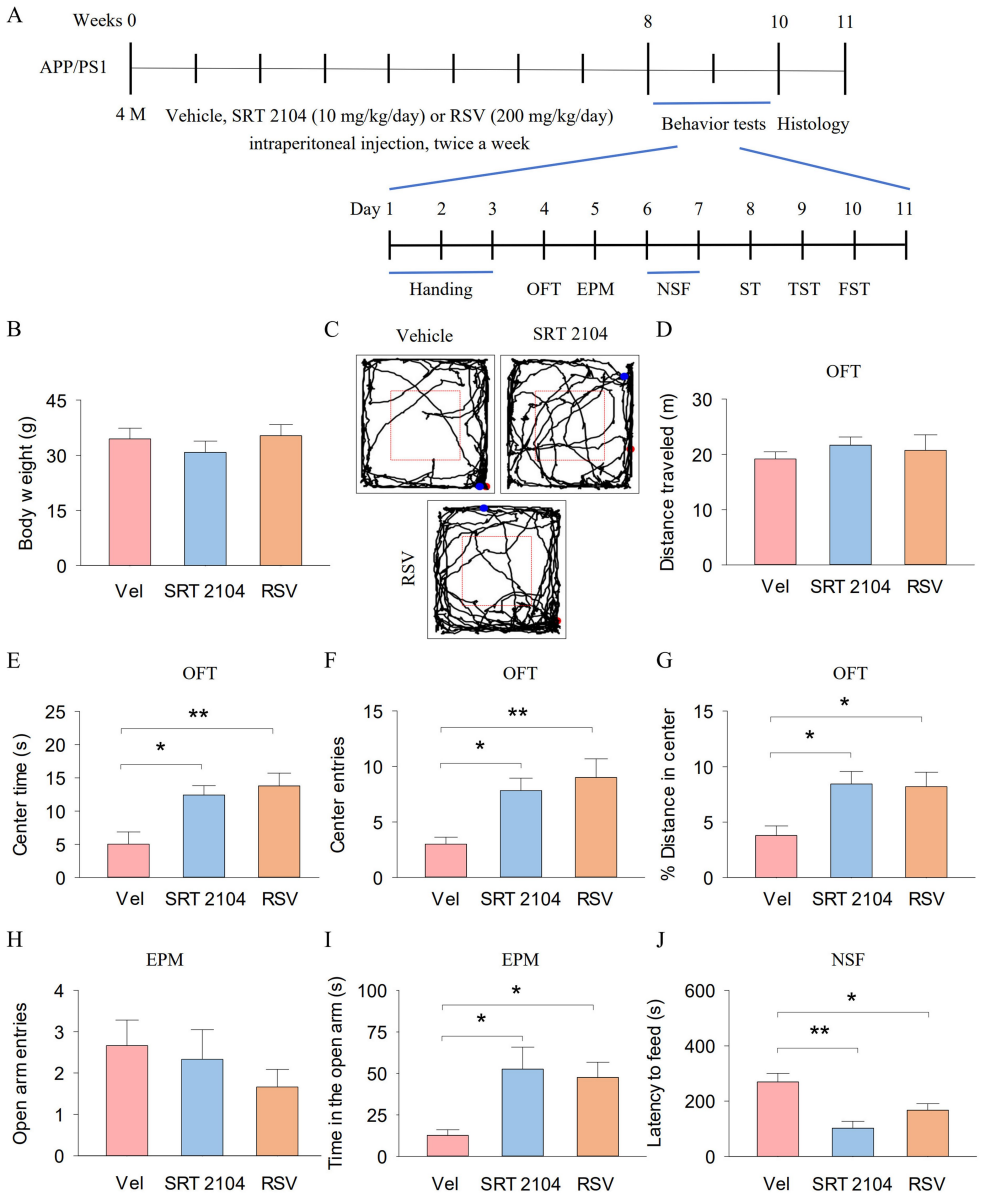
Pharmacological activation of SIRT1 ameliorate anxiety-like behavior of APP/PS1 mice. **(A)** The experimental design of the animal study. **(B)** Body weight of mice. **(C)** Representative trajectories of mice in the OFT. **(D–G)** The distance traveled **(D)**, the center time **(E)**, center entries **(F)**, and % distance in center **(G)** in the OFT for each group of mice. **(H,I)** The open arm entries **(H)** and time in the open arm **(I)** by mice in each experimental group during the EPM. **(J)** The latency to feed of mice in the NSF. n = six per group. Data are presented as mean ± SEM, One way ANOVA; *p < 0.05, **p < 0.01.

We investigate the behavioral effects of SRT2104, a selective SIRT1 activator, and Resveratrol, one of the most potent natural SIRT1 activator, on anxiety- and depression-like behaviors in APP/PS1 mice. SRT2104 (10 mg/kg/day, 0.2 mL) and Resveratrol (RSV, 200 mg/kg/day, 0.2 mL) were intraperitoneally injected twice a week from week 16 to week 24 in APP/PS1 mice (Figure 4A). Then, we measured the anxiety- and depression-like behaviors of the mice. The mouse weight was not different among the three groups (Figure 4B). Figure 4C shows representative trajectories of mice in the OFT. In the OFT, total distance traveled did not show significant differences among the three groups (Figure 4D). One-way ANOVA indicated significant effects of SIRT1 activator on the center time (*F*_2, 17_ = 7.4, *p* = 0.006; Figure 4E), center entries (*F*_2, 17_ = 6.8, *p* = 0.008; Figure 4F), and % distance in center (*F*_2, 17_ = 5.6, *p* = 0.015; Figure 4G). Tukey’s *post-hoc* tests revealed that SIRT1 activator pretreatments significantly increased the center time (SRT2104, *p* = 0.022; RSV, *p* = 0.008), center entries (SRT2104, *p* = 0.035; RSV, *p* = 0.009), and % distance in center (SRT2104, *p* = 0.032; RSV, *p* = 0.024) compared with vehicle pretreatments. In the EPM, open arm entries did not exhibit significant differences among the three groups (Figure 4H). One-way ANOVA indicated significant effects of SIRT1 activator on the time in the open arm (*F*_2, 17_ = 5.3, *p* = 0.018; Figure 4I). Tukey’s *post-hoc* tests revealed that SIRT1 activator pretreatments significantly increased the time in the open arm (SRT2104, *p* = 0.023; RSV, *p* = 0.048) compared with vehicle pretreatments. In the NSF, One-way ANOVA indicated significant effects of SIRT1 activator on the latency to feed (*F*_2, 17_ = 10.1, *p* = 0.002; Figure 4J). Tukey’s *post-hoc* tests revealed that SIRT1 activator pretreatments significantly decreased the latency to feed (SRT2104, *p* = 0.001; RSV, *p* = 0.039) compared with vehicle pretreatments.

**Figure 5:**
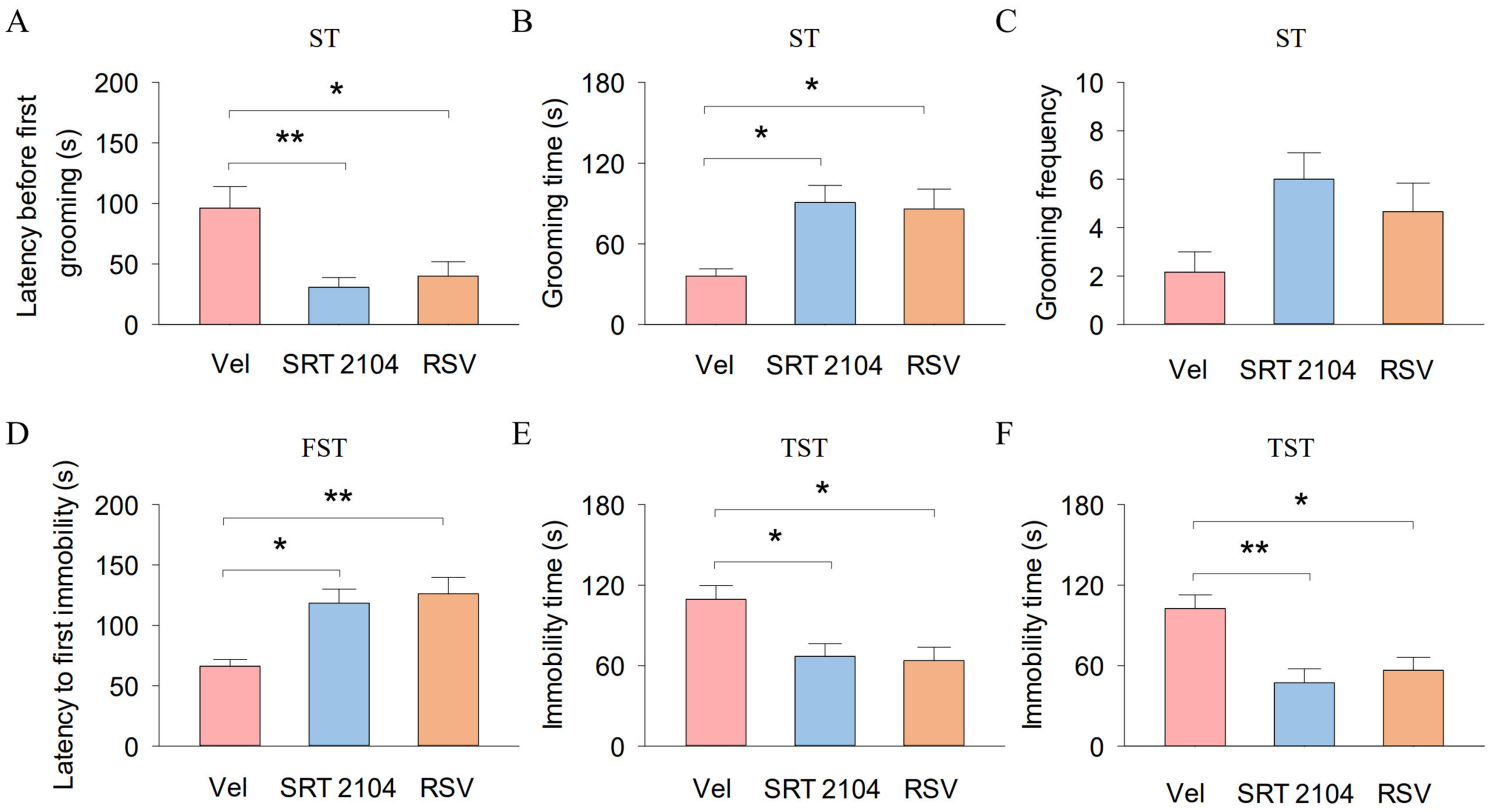
Pharmacological activation of SIRT1 ameliorate depression-like behavior of APP/PS1 mice. **(A–C)** The latency before first grooming **(A)**, grooming time **(B)**, and grooming frequency **(C)** in the ST for each group of mice. **(D,E)** The latency to first immobility **(D)** and immobility time **(E)** by mice in each experimental group during the TST. **(F)** The immobility time by mice in each experimental group during the FST. n = six per group. Data are presented as mean ± SEM, One way ANOVA; *p < 0.05, **p < 0.01.

In the ST, One-way ANOVA indicated significant effects of SIRT1 activator on the latency before first grooming (*F*_2, 17_ = 7.2, *p* = 0.006; Figure 5A) and grooming time (*F*_2, 17_ = 6.7, *p* = 0.008; Figure 5B). Tukey’s *post-hoc* tests revealed that SIRT1 activator pretreatments significantly decreased the latency before first grooming (SRT2104, *p* = 0.009; RSV, *p* = 0.022) and increased the grooming time (SRT2104, *p* = 0.012; RSV, *p* = 0.022) compared with vehicle pretreatments. However, the grooming frequency did not show significant differences among the three groups (Figure 5C). In the TST, One-way ANOVA indicated significant effects of SIRT1 activator on the latency to first immobility (*F*_2, 17_ = 9.0, *p* = 0.003; Figure 5D) and immobility time (*F*_2, 17_ = 6.6, *p* = 0.009; Figure 5E). Tukey’s *post-hoc* tests revealed that SIRT1 activator pretreatments significantly increased the latency to first immobility (SRT2104, *p* = 0.010; RSV, *p* = 0.004) and decreased immobility time (SRT2104, *p* = 0.022; RSV, *p* = 0.014) compared with vehicle pretreatments. In the FST, One-way ANOVA indicated significant effects of SIRT1 activator on the immobility time (*F*_2, 17_ = 8.7, *p* = 0.003; Figure 5F). Tukey’s *post-hoc* tests revealed that SIRT1 activator pretreatments significantly decreased immobility time (SRT2104, *p* = 0.004; RSV, *p* = 0.015) compared with vehicle pretreatments. These results suggest that hippocampal SIRT1 function is involved in anxiety- and depression-like behaviors in APP/PS1 mice. Meanwhile, hippocampal SIRT1 signaling mediates the ameliorative effect of treadmill exercise on anxiety- and depression-like behavior in APP/PS1 mice.

### 3.5 Treadmill exercise enhanced the hippocampal PGC-1α/NRF1/TFAM signaling pathway and mitochondrial biogenesis in APP/PS1 mice

**Figure 6:**
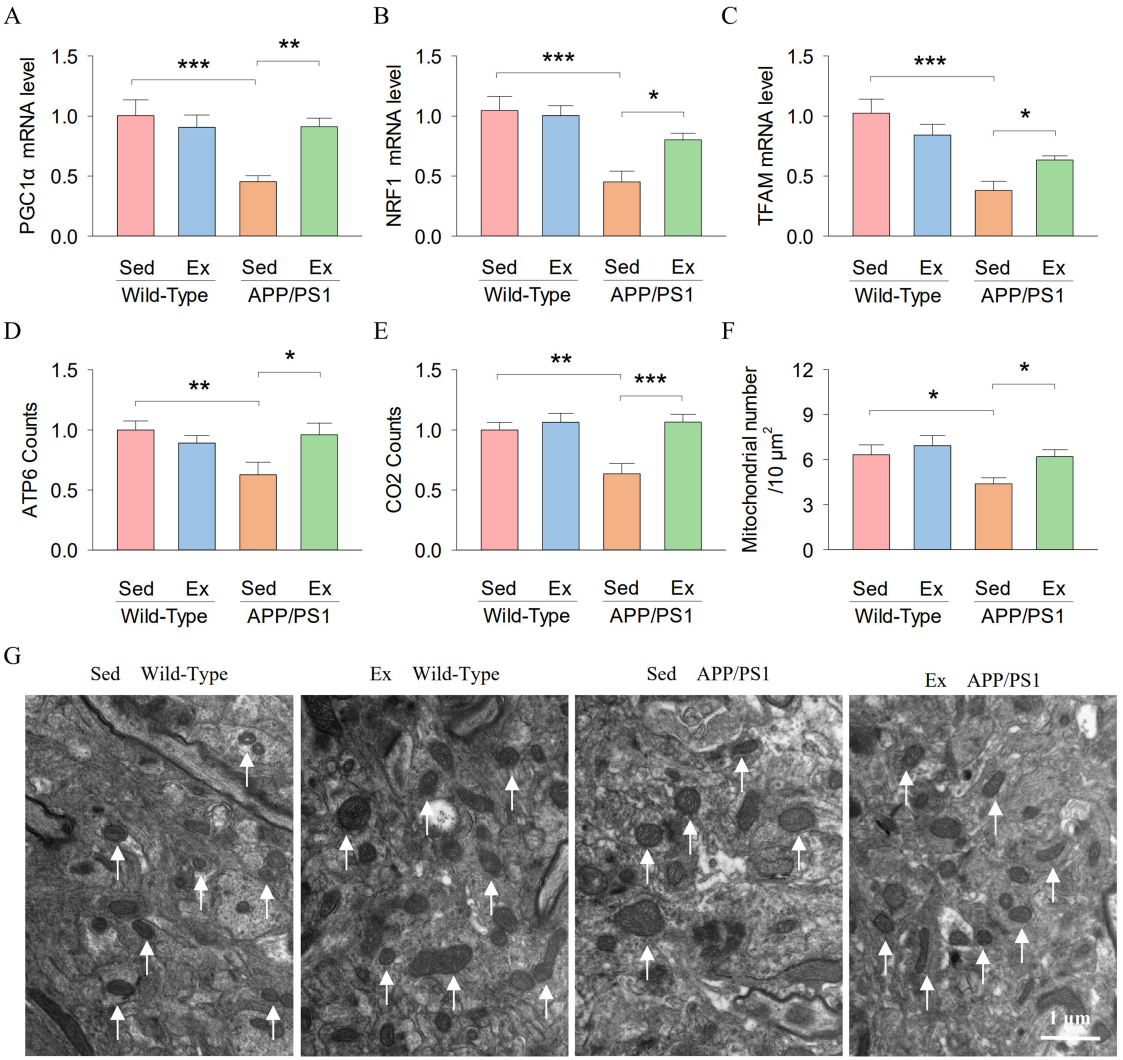
Treadmill exercise enhanced the hippocampal PGC-10/NRF1/TFAM signaling pathway and mitochondrial biogenesis in APP/PS1 mice. **(A–E)** The RT-qPCR analysis of PGCla **(A)**, NRFI **(B)**. TFAM **(C)**. ATP6 **(D)**, and CO2 **(E)** in hippocampal tissues from mice in each group. **(F)** Histogram illustrates the total number of mitochondrial. **(G)** Representative electron microscopy images of the mitochondria in the hippocampus. The arrows point to the mitochondria. Scale bar = 1 m. n = six per group. Data are presented as mean ± SEM, two way ANOVA; *p < 0.05, **p < 0.01, ***p < 0.001.

PGC-1α is a downstream target of SIRT1. It was demonstrated that PGC-1α promotes TFAM transcription and expression by activating NRF-1 (Abu et al., 2023). We further examined the levels of PGC-1α, NRF1, and TFAM in the hippocampus. Two-way ANOVA revealed that genotype and exercise had no significant main effect on the mRNA expression of PGC-1α (genotype: *F*_1,23_ = 8.3, *p* = 0.009; exercise: *F*_1,23_ = 3.7, *p* = 0.068; Figure 6A), NRF1 (genotype: *F*_1,23_ = 20.3, *p* < 0.001; exercise: *F*_1,23_ = 3.1, *p* = 0.096; Figure 6B) and TFAM (genotype: *F*_1,23_ = 25.8, *p* < 0.001; exercise: *F*_1,23_ = 0.2, *p* = 0.675; Figure 6C) in the hippocampus. However, there was a significant interaction between genotype and exercise on the mRNA expression of PGC-1α (*F*_1,23_ = 8.7, *p* = 0.008), NRF1 (*F*_1,23_ = 5.0, *p* = 0.037) and TFAM (*F*_1,23_ = 6.7, *p* = 0.018). Tukey’s *post-hoc* tests indicated that the mRNA expression of PGC-1α (*p* < 0.001; Figure 6A), NRF1 (*p* < 0.001; Figure 6B) and TFAM (*p* < 0.001; Figure 6C) in the hippocampus was significantly decreased in the AP-Sed group compared to the WT-Sed group. Treadmill exercise prevented a decrease in the mRNA expression of PGC-1α (*p* = 0.003), NRF1 (*p* = 0.011), and TFAM (*p* = 0.046) in the hippocampus in APP/PS1 transgenic mice. These data indicate that PGC-1α, NRF1, and TFAM levels are reduced in the hippocampus of APP/PS1 transgenic mice, but exercise elevated the levels of SIRT1.

The PGC-1α/NRF1/TFAM signaling pathway plays a key role in regulating mitochondrial biogenesis. Therefore, we investigated whether exercise regulates the hippocampal mitochondrial biogenesis of APP/PS1 mice. ATP6 and CO2 are subunits of the mitochondrial gene-encoded ATP synthase and complex IV, respectively. Two-way ANOVA revealed that genotype and exercise had no significant main effect on the ATP6 counts (genotype: *F*_1,23_ = 3.1, *p* = 0.092; exercise: *F*_1,23_ = 1.7, *p* = 0.201; Figure 6D) in the hippocampus. However, there was a significant interaction between genotype and exercise on the ATP6 counts (*F*_1,23_ = 6.5, *p* = 0.019), and the CO2 counts (genotype: *F*_1,23_ = 6.1, *p* = 0.022; exercise: *F*_1,23_ = 11.5, *p* = 0.003; genotype × exercise interaction: *F*_1,23_ = 6.5, *p* = 0.019; Figure 6E). Tukey’s *post-hoc* tests indicated that the ATP6 counts (*p* = 0.006; Figure 6D) and the CO2 counts (*p* = 0.002; Figure 6E) in the hippocampus were significantly decreased in the AP-Sed group compared to the WT-Sed group. Treadmill exercise prevented a decrease in the ATP6 counts (*p* = 0.013) and the CO2 counts (*p* < 0.001) in the hippocampus of APP/PS1 transgenic mice. In addition, we investigated the effects of exercise on the number of mitochondria in the mouse hippocampus. Figure 6G shows representative electron microscopy images of the mitochondria in the hippocampus. Two-way ANOVA revealed that genotype and exercise had significant main effects on the mitochondrial number in the hippocampus (genotype: *F*_1,23_ = 5.7, *p* = 0.027; exercise: *F*_1,23_ = 4.6, *p* = 0.045; Figure 6F). However, there was no significant interaction between genotype and exercise on the mitochondrial number in the hippocampus (genotype × exercise interaction: *F*_1,23_ = 1.1, *p* = 0.297). Tukey’s *post-hoc* tests indicated that the mitochondrial number in the hippocampus (*p* = 0.024; Figure 6F) was significantly decreased in the AP-Sed group compared to the WT-Sed group. Treadmill exercise prevented a decrease in the mitochondrial number in the hippocampus of APP/PS1 transgenic mice (*p* = 0.035). Together, these data suggest that treadmill exercise enhanced the hippocampal mitochondrial biogenesis of APP/PS1 mice.

### 3.6 Pharmacological activation of SIRTs enhanced the hippocampal PGC-1α/NRF1/TFAM axis and mitochondrial biogenesis in APP/PS1 mice

**Figure 7:**
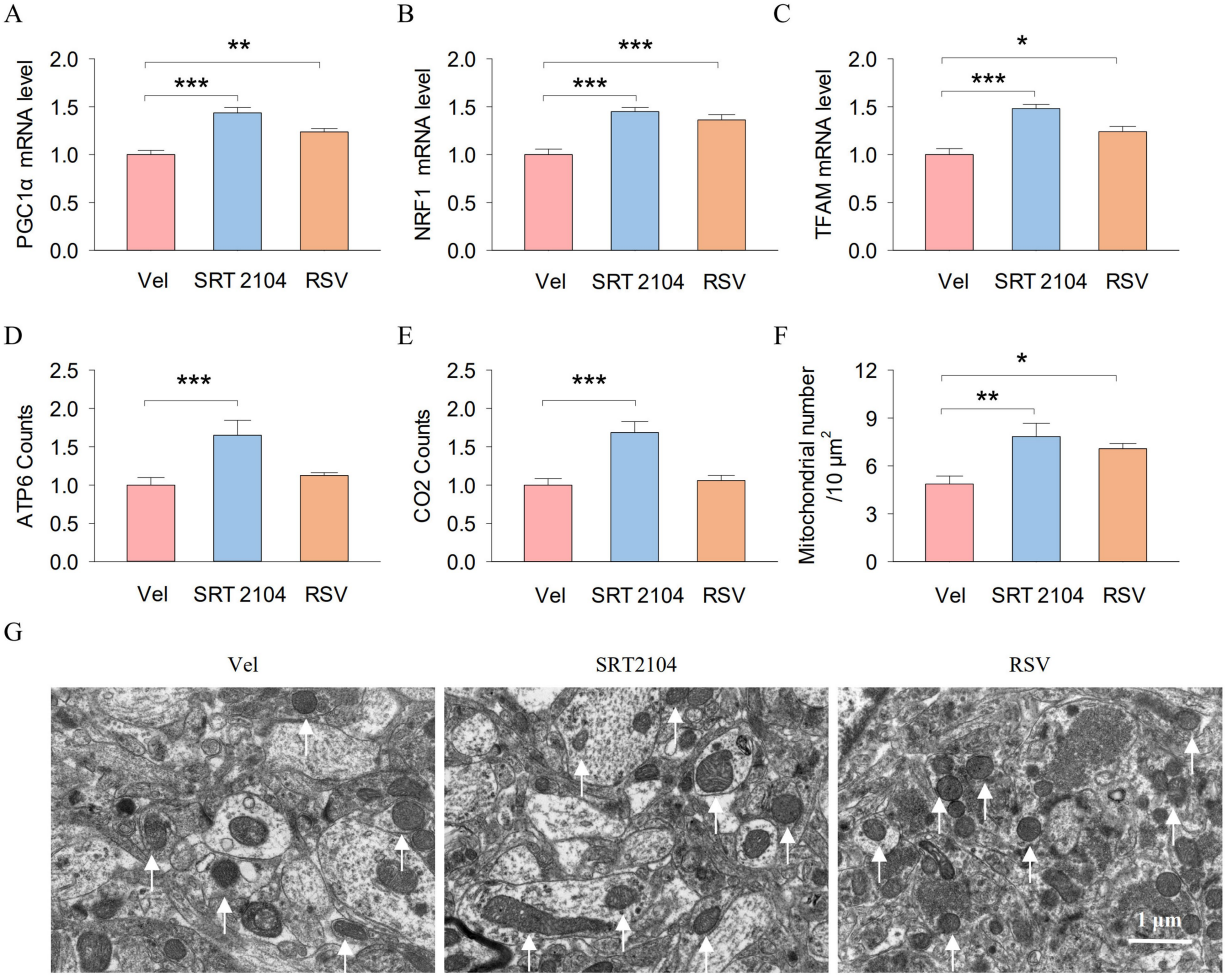
Pharmacological activation of SIRTs enhanced the hippocampal PGC-10/NRF1/TFAM axis and mitochondrial biogenesis in APP/PS1 mice. **(A–E)** The RT-qPCR analysis of PGCla **(A)**, NRFI **(B)**. TFAM **(C)**, ATP6 **(D)**, and CO2 **(E)** in hippocampal tissues from mice. **(F)** Histogram illustrates the total number of mitochondrial. **(G)** Representative electron microscopy images of the mitochondria in the hippocampus. The arrows point to the mitochondria. Scale bar =1 μm. n = six per group. Data are presented as mean ± SEM, One way ANOVA; *p < 0.05, **p < 0.01, ***p < 0.001.

Finally, we investigated the impact of pharmacological activation of SIRTs on the hippocampal PGC-1α/NRF1/TFAM axis and mitochondrial biogenesis in APP/PS1 mice. One-way ANOVA indicated significant effects of SIRT1 activator on the mRNA expression of PGC-1 (*F*_2, 17_ = 21.9, *p* < 0.001; Figure 7A), NRF1 (*F*_2, 17_ = 18.8, *p* < 0.001; Figure 7B), and TFAM (*F*_2, 17_ = 19.7, *p* < 0.001; Figure 7C) in the hippocampus. Tukey’s *post-hoc* tests revealed that SIRT1 activator pretreatments significantly increased the mRNA expression of PGC-1 (SRT2104, *p* < 0.001; RSV, *p* = 0.007), NRF1 (SRT2104, *p* < 0.001; RSV, *p* < 0.001), and TFAM (SRT2104, *p* < 0.001; RSV, *p* = 0.016) compared with vehicle pretreatments. Meanwhile, One-way ANOVA indicated significant effects of SIRT1 activator on the ATP6 counts (*F*_2, 17_ = 7.3, *p* = 0.006; Figure 7D), CO2 counts (*F*_2, 17_ = 13.4, *p* < 0.001; Figure 7E), and the number of mitochondrial (*F*_2, 17_ = 6.9, *p* = 0.007; Figure 7F) in the mouse hippocampus. Tukey’s *post-hoc* tests revealed that SIRT1 activator pretreatments significantly increased the ATP6 counts (SRT2104, *p* < 0.001), CO2 counts (SRT2104, *p* < 0.001), and the number of mitochondrial (SRT2104, *p* = 0.007; RSV, *p* = 0.044) compared with vehicle pretreatments. Figure 7G shows representative electron microscopy images of the mitochondria in the hippocampus.

## 4 Discussion

Here, we demonstrate that treadmill exercise improved anxiety- and depression-like behaviors in 6-month-old APP/PS1 mice, while also increasing hippocampal SIRT1 levels. Pharmacological activation of SIRT1 function similarly reduces anxiety- and depression-like behaviors in APP/PS1 mice. In addition, exercise promotes the activation of the PGC-1α/NRF1/TFAM axis, which is a vital element of the SIRT1 downstream signaling pathway, thereby enhancing mitochondrial biogenesis in the hippocampus of APP/PS1 model mice. Finally, we demonstrated that the pharmacological activation of SIRT1 function similarly elevated the levels of PGC1α, NRF1, TFAM, and mitochondrial biogenesis in the hippocampus of APP/PS1 mice. Taken together, these results suggest that hippocampal SIRT1 mediates the ameliorative effect of exercise on anxiety- and depression-like behaviors in APP/PS1 mice through the PGC-1α/NRF1/TFAM/mitochondrial biogenesis pathway.

Neuropsychiatric symptoms, such as depression, apathy, aggression, and psychosis, can increase the risk of AD and may lead to cognitive decline. These symptoms are now widely recognized as core features of AD. However, the predominant focus of Alzheimer’s disease (AD) research lies in cognitive decline. There has been a lack of adequate attention given to the neuropsychiatric symptoms associated with AD. We conducted a wide range of behavioral assessments to evaluate anxiety- and depression-like behaviors in APP/PS1 mice. The OFT, MBT, EPM, and NSF were used to assess whether the mice had defects in anxiety-like behavior. We found that 6-month-old APP/PS1 mice exhibited anxiety-like behaviors in the OFT, EPM, and NSF, but did not show a significant change in the MBT. In addition, the total distance traveled in the OFT did not show a significant difference, suggesting that the overall motor activity of the mice did not change. Regarding depressive-like behavior, we first utilized the ST to investigate apathy, an endophenotype of depressive behavior, and observed a decrease in grooming behavior in APP/PS1 mice. TST and FST measure the immobility of mice as an indication of despair when they are unable to escape from an aversive situation. Despair is a prominent symptom of depression. We found that APP/PS1 mice exhibited a shorter latency to first immobility and a longer immobility time in the TST. Meanwhile, the APP/PS1 mice exhibited a longer immobility time in the FST. These results indicate that APP/PS1 mice exhibited depressive-like behaviors. Anhedonia also is a core symptom of depression. Additionally, we utilized the SPT to evaluate anhedonia in mice and found no difference in the sucrose preference index between APP/PS1 and wild-type mice. It may be that anhedonia in APP/PS1 mice is age-dependent, whereas early APP/PS1 mice do not show anhedonia (Várkonyi et al., 2022). Our previous research demonstrated that pre-treatment with treadmill exercise prevented a decline in spatial working memory in mouse models of AD (Mu et al., 2022a,b). Here, we extended our previous study and further demonstrated that treadmill exercise improved anxiety- and depression-like behaviors in APP/PS1 mice. Taken together, six-month-old APP/PS1 mice exhibited anxiety- and depression-like behaviors under the OFT, MBT, EPM, ST, TST, and FST, and treadmill exercise effectively alleviated these behaviors in APP/PS1 mice.

We investigated the potential mechanisms that may underlie the reduction of anxiety- and depression-like behaviors in APP/PS1 mice induced by treadmill exercise. In AD, amyloid plaques and neurofibrillary pathology accumulate primarily in the hippocampus, cortex, and amygdala, leading to neuronal dysfunction. The hippocampus plays a significant role in regulating mood. Research shows that mood disorders, such as depression or anxiety, are causally linked to hippocampal dysfunction. Additionally, hippocampal SIRT1 signaling mediates anxiety- and depression-like behaviors in rat models of sleep deprivation and the rat chronic restraint stress model (Abe-Higuchi et al., 2016; Kang et al., 2021). Here, we have demonstrated a significant decrease in the level of SIRT1 in the hippocampal CA1 and CA3 regions of the APP/PS1 mice. Meanwhile, treadmill exercise enhanced the hippocampal SIRT1 expression in APP/PS1 mice. Previous studies indicate that pharmacological activation or local overexpression of SIRT1 in the hippocampus or BNST can reverse chronic stress-induced anxiety- and depression-like behaviors (Abe-Higuchi et al., 2016; Hu et al., 2023; Liu et al., 2019). To further elucidate the role of SIRT1 in improving anxiety- and depression-like behaviors in APP/PS1 mice through exercise, this study examined the impact of SIRT1 agonists on anxiety and depressive behaviors in APP/PS1 mice. We found that pharmacological activation of SIRT1 also alleviates anxiety- and depression-like behaviors in APP/PS1 mice. These results indicate that hippocampal SIRT1 mediates the ameliorative effect of treadmill exercise on anxiety- and depression-like behavior in APP/PS1 mice.

PGC-1α is a downstream target of SIRT1. Several studies have shown that deacetylation of PGC-1α is dependent on SIRT1 activity, which increases the transcriptional activity of PGC-1α (Amat et al., 2009; Gurd, 2011). PGC-1α functions as a transcriptional coactivator that stimulates the activity of NRF-1 and TFAM to regulate mitochondrial biogenesis (Cardanho-Ramos and Morais, 2021; Zhao et al., 2020). Mitochondrial biogenesis is a complex biological process that regulates mitochondrial DNA replication, gene transcription, and the formation of new mitochondria, thereby maintaining cellular homeostasis. Previous studies have shown that the levels of PGC-1α, NRF1, and TFAM in the hippocampus of AD patients and animal models are significantly reduced, indicating impaired mitochondrial biogenesis (Sheng et al., 2012; Singulani et al., 2020). Consistent with previous studies, this study documented a significant reduction in the expression levels of PGC-1α, NRF1, TFAM, ATP6, CO2, and mitochondrial content in the hippocampus of APP/PS1 mice. However, treadmill exercise intervention can effectively increase the levels of PGC-1α, NRF1, TFAM, ATP6, CO2, and mitochondrial content in the hippocampus of APP/PS1 mice. Therefore, exercise can enhance mitochondrial biogenesis in the hippocampus of APP/PS1 model mice. In addition, we further examined the effects of pharmacological activation of SIRT1 on mitochondrial biogenesis and found that such activation also increases the levels of PGC-1α, NRF1, TFAM, ATP6, CO2, and mitochondrial content in the hippocampus of APP/PS1 mice. In summary, exercise may enhance mitochondrial biogenesis in the hippocampus of APP/PS1 mice through the activation of the SIRT1/PGC-1α/NRF1/TFAM axis. An increasing number of studies suggest that disturbances in mitochondrial biogenesis are involved in the development and progression of anxiety and depression (Yan et al., 2023; Yin et al., 2022). In total, hippocampal SIRT1 regulates the SIRT1/PGC-1α/NRF1/TFAM/mitochondria biogenesis axis to mediate the ameliorative effect of treadmill exercise on anxiety- and depression-like behaviors in APP/PS1 mice. This study presents innovative perspectives on behavioral and pharmacological interventions for the prevention and treatment of AD.

## Data Availability

The original contributions presented in the study are included in the article/supplementary material, further inquiries can be directed to the corresponding authors.
